# The clinical outcome of postoperative radiotherapy using hybrid planning technique in left breast cancer after breast‐conserving surgery

**DOI:** 10.1002/cam4.5358

**Published:** 2022-10-17

**Authors:** Ting‐Na Wei, Hui‐Ling Yeh, Jia‐Fu Lin, Chih‐Chiang Hung

**Affiliations:** ^1^ Department of Radiation Oncology Taichung Veterans General Hospital Taichung Taiwan; ^2^ Department of Radiation Physics Taichung Veterans General Hospital Taichung Taiwan; ^3^ Department of Breast Surgery Taichung Veterans General Hospital Taichung Taiwan

**Keywords:** breast cancer, prognosis, radiotherapy, survival

## Abstract

**Background:**

The purpose of this study is to observe the preliminary clinical outcome and acute toxicity of hybrid intensity modulated radiotherapy and volumetric modulated arc therapy planning technique with simultaneous integrated boost (SIB).

**Methods:**

From November 2015 to December 2018, 149 female patients with left‐side breast cancer who underwent adjuvant radiotherapy with hybrid IMRT and VMAT planning technique with SIB were reviewed retrospectively. The primary endpoint was acute toxicities and the secondary endpoints were local recurrence‐free survival (LRFS), distant metastasis‐freesurvival (DMFS), disease‐free survival (DFS), and overall survival (OS).

**Results:**

The median age was 52 years old and median follow‐up was 43.4 months. Eighty‐six percent of patients had acute grade 0 to grade1 dermatitis and 14% had grade 2 dermatitis. No acute radiation pneumonitis, esophagitis, or cardiovascular events were recorded during follow‐up. The 3‐year LRFS, DMFS, DFS, and OS rates were 95.1%, 95.1%, 90.3%, and 97.9%, respectively. The subgroup analysis revealed that patients with lymphovascular invasion had more local recurrence rate and worse DFS rate. Patients with advanced N stage had the trend of worse DMFS.

**Conclusion:**

In conclusion, the hybrid IMRT and VMAT technique is feasible, safe and has less acute radiation related toxicities in SIB postoperative radiotherapy for left‐sided breast cancer.

## Graphical abstract

This study was to observe the preliminary clinical outcome and acute toxicity of hybrid intensity modulated radiotherapy (IMRT) and volumetric modulated arc therapy (VMAT) planning technique with simultaneous integrated boost (SIB). The result showed the hybrid technique was feasible, safe and had less acute radiation related toxicities in SIB postoperative radiotherapy for left‐sided breast cancer.

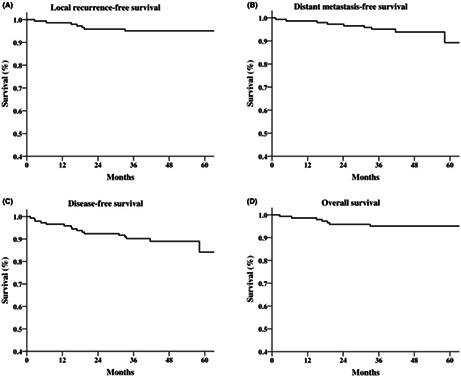

## 1 INTRODUCTION

The purpose of breast adjuvant radiotherapy is to reduce the local recurrence rate and the mortality.^1^, ^2^ In previous randomized clinical trials, breast‐conserving surgery plus postoperative radiotherapy has been proven to achieve an equivalent survival rate to that of mastectomy in the patients with early breast cancer.^2^, ^3^, ^4^ Most patients with early breast cancer are cured after a series of treatments. The hazard of radiotherapy should be taken into consideration as the patient can be expected to have a long survival.

Two major organs at risk (OAR) in breast radiotherapy are the heart and the lung. Radiation‐induced cardiovascular toxicities include coronary artery disease (CAD), arrhythmia, pericarditis, cardiomyopathy, valvular dysfunction, and heart failure.^5^ The long‐term follow‐up in some trials have shown that radiotherapy can also increase the risk of morbidity and mortality from coronary heart disease.^6^ The adjuvant radiotherapy complications include radiation pneumonitis and, later on, lung fibrosis and subsequent primary lung cancer.^7^ The absolute risks of the adjuvant radiotherapy complications depend on the volume of the normal organs receiving doses. To reduce radiation doses to the volume of normal tissue and the scatter radiation dose while still maintaining high quality in homogeneity and conformity for the treatment targets is the major concern in designing the treatment plan.

Modern radiotherapy techniques such as conformal three‐dimensional radiotherapy (3D‐CRT), intensity‐modulated radiotherapy (IMRT), image‐guided irradiation (IGRT), and volumetric modulated arc therapy (VMAT) help us optimize the treatment plan while decreasing the incidences of complications. Since 2015, we have applied hybrid techniques which incorporating IMRT and VMAT planning techniques, with two tangential fields plus two half coplanar arcs, in the treatment of patients with left‐side breast cancer. A previous dosimetry comparison study showed that this technique was feasible for whole‐breast irradiation.^8^ Our previous study showed that hybrid technique could achieve better plan dosimetry, quality, and treatment efficacy when compared to fixed‐field IMRT or pure‐VMAT.^8^

An additional radiation boost dose to the surgical bed was found to improve local tumor control in the previous studies. The EORTC boost‐trial revealed that an additional radiation boost could reduce the local recurrence rate effectively.^1^ Compared with sequential radiation boost dose to the tumor cavity, simultaneous integrated boost (SIB) not only provides a higher dose in the tumor bed and a lower dose in the remaining breast, but also has a shorter treatment duration. The dosimetric advantages to the target coverage and higher radiation dose gradient per fraction to OAR could be achieved by SIB.^9^

The results of treatment planning dosimetry comparisons with various hybrid techniques have been reported in several series, but data on the treatment outcomes are sparse.^10^ The hybrid planning technique by combining IMRT and VMAT became our daily routine planning for left side breast cancer since 2015. The aim of this retrospective study was to evaluate the preliminary clinical outcome of disease control and acute toxicity in a cohort of left‐side breast cancer patients receiving postoperative radiotherapy with SIB to the tumor bed using hybrid planning technique (IMRT plus VMAT). In 2020, this retrospective study was approved by our Institutional Review Board and the Research Ethical Committee (No: CE20164A). The committee approved us to waive the documentation of informed consent.

## 2 MATERIALS AND METHODS

### 2.1 Patient selection

From November, 2015 to December, 2018, a total of 149 female patients with left‐side breast cancer who were treated at the Department of Radiation Oncology in Taichung Veterans General Hospital were enrolled in this retrospective study. All patients underwent breast‐conserving surgery then received adjuvant radiotherapy. Some of the patients with positive result in sentinel lymph node biopsy (SLNB) underwent axillary lymph node dissection (ALND) and regional lymph node irradiation. The surgical pathology reports and medical records were reviewed. The cancer staging was performed according to the 7th edition of the American Joint Committee on Cancer. Pathological characteristics of breast including tumor pathological type, size, molecular type, lymphovascular invasion (LVI), perineural invasion (PNI), T‐stage, axillary nodal status and margin status were all recorded. Treatment details of neo‐adjuvant or adjuvant systemic treatment were collected. The exclusion criteria were the patients with right side breast cancer, age ≦ 20 years‐old, those received total mastectomy, and those had incomplete radiotherapy.

### 2.2 Treatment planning

A customized vacuum bag was made for every patient for immobilization of the treatment position. To acquire a series of Computed tomography (CT) images of our patients for contouring, CT simulation was performed in the supine position with left arm‐up position on a customized vacuum bag. A set whole chest +/− lower neck CT scan images with 5‐mm slice thickness was extracted for tumor and organ at risk (OAR) contouring according to the treatment protocol of our department. According to our treatment protocol of CT scan slices thickness before 2015, 4 mm thickness of each CT scan slice is suggested for head and neck cancer; 5 mm for chest and abdomen organ; and 1 cm for total body irradiation. This protocol was based on the Photon Treatment Collaborative Work Group's recommendation. The contouring guidelines for the clinical target volume (CTV) of the whole left breast and regional lymph nodes were based on the Radiation Therapy Oncology Group (RTOG) Breast Cancer Atlas. The regional lymph node including ipsilateral infraclavicular fossa and supraclavicular fossa were contoured to the CTV in the patients who had pathologic proved tumor invasion in the lymph nodes after in SLNB and ALND. When the tumor was located centrally or medially with lymph node involvement, the internal mammary node chain (IMC) was also included in the CTV. The CTV of whole breast expanded about 0.5 cm to become the planning target volume (PTV) but this CTV expansion was restricted to the tissue within 3 mm from the skin. The CTV of boost dose was defined as the surgical clip, postoperative seroma or tumor cavity in CT simulation. A 5‐mm margin was added to the CTV of boost to obtain the PTV of boost dose without exceeding the CTV of left breast. The prescribed total radiation dose to the whole breast was 1.8 Gy per fraction to a total radiation dose of 50.4 Gy in 28 fractions and the simultaneous integrated boost dose of 2.214 Gy per fractions was delivered to the tumor bed up to 62.0 Gy in 28 fractions.

The treatment planning system was the Varian Eclipse. All plans were designed to deliver 6 MV photon beams from a Varian iX linear accelerator equipped with a Millennium Multileaf collimator (MLC) with 120 leaves. The hybrid planning technique was designed by combining 75% of the prescribed radiation doses from 2 tangential IMRT fields plus 25% of the prescribed radiation doses from 2 partial coplanar arcs. Detail of planning technique was described in our previous study.^8^ The treatment plans were optimized according to Emami's paper.^11^ Ninety‐five percent of PTV was covered by 95% prescribed dose. The normal tissue dose constraints were based on Quantitative Analyses of Normal Tissue Effects in the Clinic (QUANTEC).^12^ The constraints for the heart were mean heart dose <8 Gy, V_25Gy_ of heart<10% and the D_0.03cc_of left anterior descending corartery dose (EQD23 Gy) <45.5 Gy. The constraints for the ipsilateral lung were ipsilateral lung mean dose <15 Gy, V_20Gy_ of lung <20% and V_5Gy_ of lung <60%.

### 2.3 Follow‐up and outcome

#### 2.3.1 Toxicity evaluation

The acute toxicities grading were stratified according to the Common Terminology Criteria for Adverse Events (version 4.0). During the radiotherapy course, patients visited the radiation oncologist in the clinic every week to monitor the side effects. After completing adjuvant radiotherapy, all of the patients were followed up at our clinic within 3 months to ensure that the acute toxicity had been resolved and then every 6 months to 5 years. Breast sonography, abdominal sonography, and chest X‐ray were arranged every 6 months, while breast mammography was arranged annually for 5 years. Whole body bone scans, chest or abdominal CT and positron emission tomography (PET) scan were optional, depending on the clinical conditions. Radiation pneumonitis, esophagitis, and heart events were assessed after the treatment using patients' medical records. The highest‐grade of acute toxicities were recorded for further analysis.

#### 2.3.2 Plan evaluation and dosimetry parameters

In the dosimetric analysis, the following data of OAR were recorded from dose–volume histograms, including ipsilateral lung mean dose, V_20 Gy_ and V_5 Gy_; heart V_25 Gy_; contralateral lung mean dose and V_5 Gy_; contralateral breast mean dose andV_5 Gy_. We used conformity index (CI) and homogeneity index (HI) to evaluate the quality of our plan. The CI was carried out using the following formula:

$$\mathrm{CI}=\frac{V_{\mathrm{PTV},\mathrm{ref}}}{V_{\mathrm{PTV}}}\times \frac{V_{\mathrm{PTV},\mathrm{ref}}}{V_{\mathrm{ref}}}$$

The homogeneity index (HI) formula as:

$$\mathrm{HI}=\frac{D_{2\%}-D_{98\%}}{D_{50\%}}$$

The homogeneity index of SIB and PTV (excluding the high‐dose region of SIB) were calculated separately. The calculation of CI and HI were based on the definition of the International Commission on Radiation Units and Measurements.

#### 2.3.3 Statistics analysis

The follow‐up time was defined from the time of the completion of radiotherapy to the latest follow‐up records or death. The definitions of local recurrence‐free survival (LRFS), distant metastasis‐free survival (DMFS), disease‐free survival (DFS), and overall survival (OS) were the period from the last time of irradiation to the date of ipsilateral breast or regional lymph node recurrence, first time of distant metastasis, any events comprising local recurrence, distant metastasis, second cancers, and death from any cause and death from any cause, respectively. The primary endpoints were acute and late toxicities. The secondary endpoints were the clinical outcome of LRFS, DMFS, DFS, and OS.

The survival analysis of LRFS, DMFS, DFS, and OS was calculated by Kaplan–Meier methods. Univariate and multivariate analysis were performed by using log‐rank test and Cox regression analysis to find out the potential prognostic factors. The prognostic factors included: age (≥50 yr vs. <50 yr), T‐stage (T0–T1 vs. T2–T4), N‐stage (N0 vs. N1–N2), LVI (presence vs. absence), PNI (presence vs. absence), tumor estrogen receptor (ER) status (positive vs. negative), neoadjuvant therapy (Yes vs. No), and adjuvant therapy, including hormone therapy (Yes vs. No). A *p* value < 0.05 was considered to be statistically significant. The statistical analyses were performed using SPSS software, version 22.0 (IBM Corporation, USA).

## 3 RESULTS

### 3.1 Patients' characteristic and clinical outcomes

The median follow‐up time of the 149 patients was 43.4 months. Table 1 lists the patients' characteristics. All patients were female and had left‐side breast cancer. The median age was 52 years old. Among all of the patients, infiltrating ductal carcinoma accounted for 76.5% of cases and 59.1% were classified as luminal subtypes. All the resection margins were negative after breast‐conserving surgery. Seventy‐six patients (51.0%) received neoadjuvant systemic treatments and 135 patients (90.6%) received adjuvant systemic treatments, including hormone therapy. The median radiotherapy duration was 40 days. Fifty‐four patients (36.2%) had regional lymph node irradiation.

**TABLE 1:** Patients' characteristics

Factors	Number or median	Percentage or IQR
Follow‐up time (months)	43.4	35.7–53.5
Age	52	45–57
Tumor size (cm)		
Sonography	2.1	1.5–2.8
Pathologic	1.1	0.4–1.9
Histology		
DCIS	24	16.1
IDC	114	76.5
Others	11	7.4
Molecular subtype		
Luminal	88	59.1
HER2	16	10.7
TNBC	20	13.4
DCIS or others	25	16.8
Tumor grade		
Grade 1	11	7.4
Grade 2	67	44.9
Grade 3	44	29.5
Not graded	5	3.4
DCIS	22	14.8
Angiolymphatic invasion		
No	122	81.9
Yes	24	16.1
Unknown	3	2.0
Perineural invasion		
No	134	89.9
Yes	11	7.4
Unknown	4	2.7
Clinical stage		
0	25	16.8
IA	42	27.5
IB	1	0.7
IIA	39	26.2
IIB	33	22.1
IIIA	6	4.0
IIIB	1	0.7
IIIC	1	0.7
Not applicable	2	1.3
Pathologic stage		
0	20	13.4
IA	32	21.5
IB	1	0.7
IIA	13	8.7
IIB	5	3.4
IIIA	1	0.7
yp0/y0	19	12.8
yIA	25	16.8
yIB	6	4.0
yIIA	15	10.1
yIIB	5	3.4
yIIIA	4	2.7
yIIIB	0	0.0
yIIIC	1	0.7
yIV	1	0.7
Not applicable	1	0.7
Systemic treatment		
Neoadjuvant	76	51.0
Adjuvant (include hormone)	135	90.6

Abbreviations: DCIS, ductal carcinoma in situ; HER2, Human Epidermal Growth Factor Receptor 2; IDC, infiltrative ductal carcinoma; IQR, interquartile range; TNBC, triple negative breast cancer.

With regard to clinical outcome, ipsilateral breast recurrence was observed in 4 of the 149 patients (2.6%) and 2 patients (1.3%) had regional lymph node recurrence. Nine (6.0%) patients had distant metastasis during follow‐up. Among the patients with distant metastasis, the most frequent site was bone. Death was recorded in 5 patients (3.4%), of whom 4 (2.7%) died due to breast cancer progression.

**FIGURE 1:**
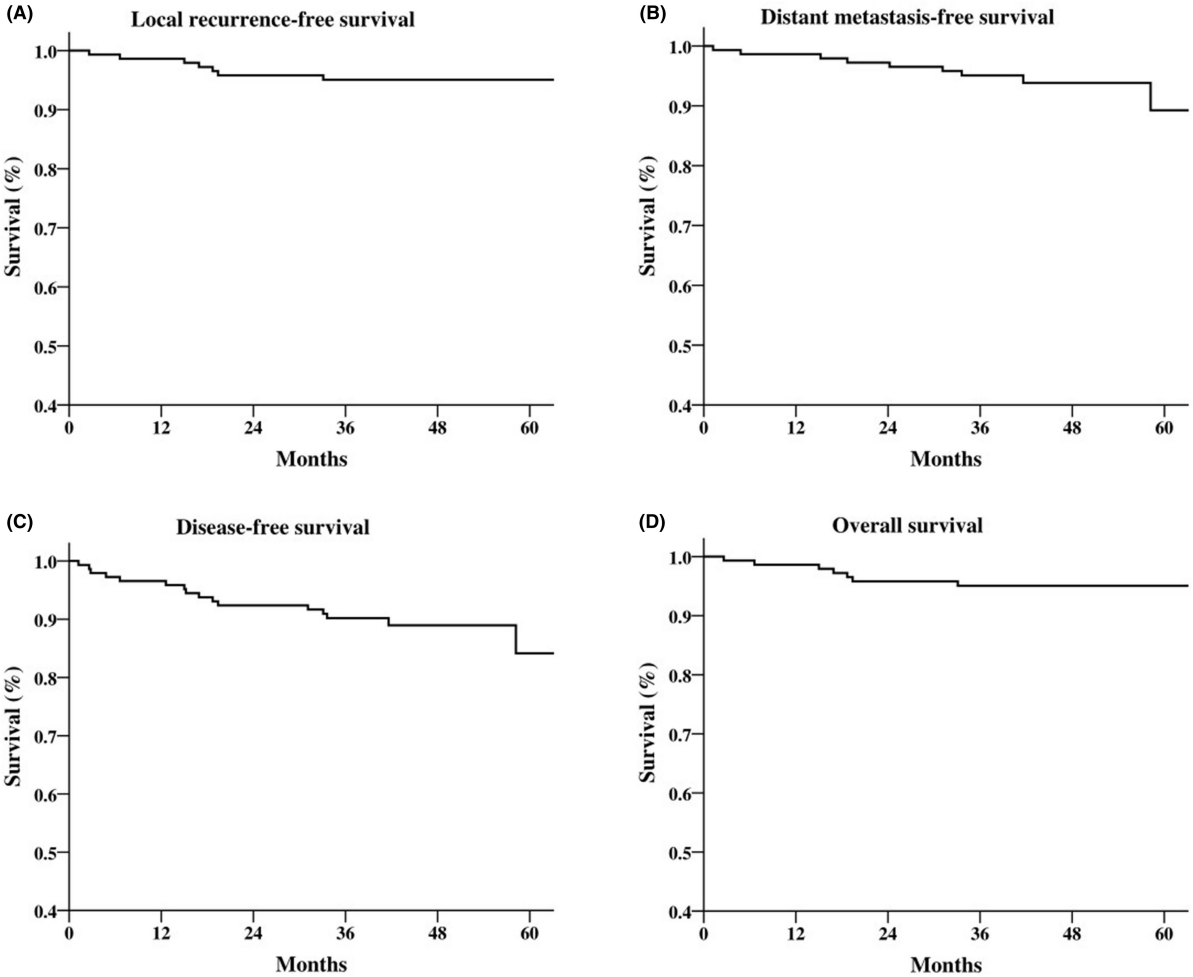
Survival curve of (A) local recurrence‐free survival, (B) distant metastasis‐free survival, (C) disease‐free survival, and (D) overall survival of 149 patients with left‐side breast cancer treated with hybrid intensity modulated radiotherapy and volumetric modulated arc therapy with simultaneous integrated boost technique

The 3‐year LRFS, DMFS, DFS, and OS rates were 95.1%, 95.1%, 90.3%, and 97.9%. The Figure 1 demonstrated the survival curve of our patients.

### 3.2 Toxicity

The majority of the patients had mild acute toxicity (grade 0 to 1) in the skin (86%). There was no grade 3 radiation dermatitis observed. Two patients complained of symptomatic cough temporarily (grade 1 radiation pneumonitis) and four patients had symptomatic dysphagia (grade 1 esophagitis) during radiotherapy. Neither grade 2 or above radiation pneumonitis nor esophagitis was noted in any patient. No cardiovascular events were noted during follow‐up. Further details are listed in Table 2.

**TABLE 2:** Acute toxicity

	Grade 0	Grade 1	Grade 2
Dermatitis	5 (3%)	123 (83%)	21(14%)
Cough	147 (99%)	2 (1%)	0 (0%)
Pneumonitis	149 (100%)	0 (0%)	0 (0%)
Dysphagia	145 (97%)	4 (3%)	0 (0%)
Esophagitis	149 (100%)	0 (0%)	0 (0%)

*Note*: Grading according to Common Terminology Criteria for Adverse Events (version 4.0).

### 3.3 Dosimetric

The median values of ipsilateral lung V_20Gy_ and V_5Gy_ were 15.2% and 53.6%, respectively. The median value of ipsilateral lung mean dose for all patients was 1039.3 cGy. The median value of heart V_25Gy_ was 3.0%. The radiation doses of normal tissue were all within the constraints based on QUANTEC. Regarding the quality of the plan in terms of homogeneity and conformity, the median CI value was 0.85. The median HI of the boost dose was 9.16% and HI of PTV (excluding SIB dose) was 25.25%. Dosimetric parameters are shown in Table 3.

**TABLE 3:** Dosimetric parameters

Factors	Median	IQR
Ipsilateral lung		
Mean dose (cGy)	1039.3	976.8–1118
V_20_ (%)	15.2	13.5–16.7
V_5_ (%)	53.6	48.6–58.6
Heart		
V_25_ (%)	3.0	1.9–5.0
Contralateral lung		
Mean dose (cGy)	355.1	300.3–434.2
V_5_ (%)	15.4	9.5–25.4
Contralateral breast		
Mean (cGy)	264.7	215–303.4
V_5_ (%)	6.9	3.7–10.4
CI	0.85	0.82–0.88
HI of SIB (%)	9.16	7.86–11.10
HI of PTV exclude SIB (%)	25.25	20.51–31.47

Abbreviations: CI, conformity index; HI, homogeneity index; IQR, interquartile range; PTV, planning target volume; SIB, simultaneous integrated boost.

$$\mathrm{CI}=\frac{V_{\mathrm{PTV},\mathrm{ref}}}{V_{\mathrm{PTV}}}\times \frac{V_{\mathrm{PTV},\mathrm{ref}}}{V_{\mathrm{ref}}}.$$

$$\mathrm{HI}=\frac{D_{2\%}-D_{98\%}}{D_{50\%.}}.$$

### 3.4 Subgroup analysis

The results of the multivariate analysis with Cox proportional hazard model are shown in Table 4. No predictors of OS could be found due to few death events. Patients with LVI had more local recurrence (HR = 5.98, *p* = 0.028, 95% CI: 1.22–29.33) and worse DFS (HR = 3.32, *p* = 0.027, 95% CI: 1.15–9.61). Patients with advanced N stage had a trend of worse DMFS (HR = 10.53, *p* = 0.028).

**TABLE 4:** Results of multivariate analyses of factors

Predictors	HR	95%CI	*p*‐value
LRFS			
LVI	5.98	1.22–29.33	0.028*
PNI	2.34	0.41‐13.37	0.339
DMFS			
LVI	2.77	0.73–10.53	0.135
N stage	10.53	1.28–86.24	0.028*
DFS			
LVI	3.32	1.15–9.61	0.027*
N stage	2.86	0.66–12.39	0.160
Neoadjuvant therapy	3.17	0.60–16.63	0.173

Abbreviations: CI, confidence interval; DFS, disease‐free survival; DMFS, distant metastasis‐free survival; HR, hazard ration; LRFS, local recurrence‐free survival; LVI, lymphovascular invasion; PNI, perineural invasion.

* *p* < 0.05.

## 4 DISCUSSION

Using two different techniques of conformal therapy combine in a treatment plan is not a new concept, but the previous studies of various hybrid techniques focused on dosimetry comparisons.^8^, ^13^, ^14^ After searching previous papers, our retrospective study is the first investigation to demonstrate the clinical outcomes of patients treated with hybrid IMRT and VMAT planning techniques. We believe our method has advantages in terms of dosimetry as well as its clinical application.

First, the minimum low‐dose constraints to OARs could be achieved by hybrid IMRT and VMAT with simultaneous integrated boost dose, especially in the lung and heart. In adjuvant whole breast irradiation, the lung is the primary and critical organ of concern. In our study, the D_mean_, V_5Gy_, and V_20Gy_of ipsilateral lung were 1039.3 cGy, 53.6% and 15.2%, which were less than 1136.9 cGy, 57.2% and 17.1% reported by Chen et al. for IMRT with SIB technique.^15^ The three aforementioned dosimetric parameters are essential predictors of radiation‐induced lung toxicity in most previous study. Goldman et al. revealed incidence of symptomatic radiation pneumonitis was significantly decreased if the V_20Gy_ of the ipsilateral lung was less than 30%.^16^ The heart is the most important organ to protect especially in left‐side breast radiation therapy. A population‐based study by Beaton et al. showed that the long term radiation‐induced cardiac death was very low in the patient who had adjuvant radiotherapy to the breast or chest wall if the radiation dose of 2500 cGy to the heart <5% (V_25Gy_ <5%).^17^ The median V_25Gy_ in our study was 3.0%. Thus, the expected lung and cardiac toxicity associated with irradiation in our hybrid technique should be reasonably low. However, longer period of follow up was needed to approve this expectation. The quality of the treatment planning was evaluated by conformity index and homogeneity index. In our study, the median value of CI was 0.85. The median HI of boost dose was 9.16% and HI of PTV (excluding SIB dose) was 25.25%. However, comparisons with previous studies may not be meaningful due to the different formulas used to perform calculations.

Regarding the prescribed dose, we chose the SIB technique rather than the sequential boost, not only due to the shorter treatment time, which can reduce the time burden to the patients, but also to provide better homogeneity in dose distribution. There are many delivery methods for SIB, such as 3D‐CRT, IMRT, helical tomotherapy, and VMAT. A dose comparison done by Aly et al. showed SIB with VMAT technique is the most balanced choice when considering optimized target coverage, homogeneity and dose scatter to the OARs.^18^ Furthermore, the EORTC boost trial demonstrated significantly lower rates of local recurrence and reduced the number of salvage mastectomies.^1^ After adding the boost dose, the possibility of an increased risk of acute toxicity is a concern. Hamilton et al. conducted a systematic review to assess acute toxicity with concurrent tumor bed boost regimes and the result showed 43–71% of patients had acute grade 2 dermatitis toxicities.^19^ In comparison with previous studies, the incidence of grade 2 dermatitis in our study was much lower. The reason for less severity of acute radiation dermatitis in our study may closely related to the shorter treatment duration by using SIB in our hybrid IMRT and VMAT treatment technique.^20^

Less acute side effect was noted from our hybrid technique when compared with other reports using IMRT or conventional therapies. In our study, eighty‐six percent of patients had acute grade 0–1 dermatitis, 14% had grade 2 dermatitis and no grade 3 was observed. Pigonol et al reported 31.2% of moist desqumation (grade 2) from those treated by IMRT for whole breast radiotherapy.^21^ Another retrospective study comparing the long term outcome between IMRT and conventional tangential radiotherapy showed 42% of the patient had developed grade 2–3 radiation dermatitis and higher incidence was found from the conventional radiotherapy.^22^ Our result of acute radiation dermatitis was much better than previous articles.

The clinical outcomes of breast radiotherapy using a hybrid IMRT and VMAT planning technique have rarely been published. Only few series have reported clinical data on IMRT with SIB technique. McDonald et al. demonstrated the 3‐year locoregional control rate was 97.1% with an OS of 97%.^23^ Fiorentino et al. also demonstrated an excellent 3‐year locoregional control of 97.2% in elder patients.^24^ Lee et al. compared the outcome of SIB delivery with IMRT or tomotherapy and the outcome of the IMRT group showed the 5‐year LRFS was 94.9%, the 5‐year DMFS was 91.4%, and the 5‐year OS was 94.3%.^25^ Our results verify that the hybrid IMRT and VMAT technique with SIB can achieve 3‐year LRFS, 3‐year DMFS, DFS, and OS rates were 95.1%, 95.1%, 90.3%, and 97.9%.

The limitation of this study was the retrospective nature of the investigation, which may be biased due to incomplete data collection. More long‐term follow‐up studies or prospective studies are needed.

## 5 CONCLUSION

In conclusion, the hybrid IMRT and VMAT technique is feasible, safe and has less acute radiation related toxicities in SIB postoperative radiotherapy for left‐sided breast cancer.

## AUTHOR CONTRIBUTIONS

**Ting‐Na Wei:** Data curation (equal); formal analysis (equal); methodology (equal); writing – original draft (lead). **Hui‐Ling Yeh:** Conceptualization (equal); data curation (equal); methodology (equal); writing – review and editing (lead). **Jia‐Fu Lin:** Conceptualization (equal); methodology (equal). **Chih‐Chiang Hung:** Conceptualization (equal); data curation (equal).

## CONFLICT OF INTEREST

All authors had no conflict of interest to declare.

## ETHICS APPROVAL STATEMENT

The trial was approved by the Institutional Review Board I & II of Taichung Veterans General Hospital and conducted according to the principles of the Declaration of Helsinki and the Guidelines for Good Clinical Practice. The Clinical trial registration number: TCVGH‐IRB No. CE20164A. This was a retrospective study and the institutional review board approved us to waive the documentation of informed consent. We ensured the anonymity of the patients' data in analysis.

## Data Availability

The data that support the findings of this study are available on request from the corresponding author. The data are not publicly available due to privacy or ethical restrictions.
